# Brief psychotherapy administered by non-specialised health workers to address risky substance use in patients with multidrug-resistant tuberculosis: a feasibility and acceptability study

**DOI:** 10.1186/s40814-020-00764-1

**Published:** 2021-01-19

**Authors:** Gregory L. Calligaro, Zani de Wit, Jacqui Cirota, Catherine Orrell, Bronwyn Myers, Sebastian Decker, Dan J. Stein, Katherine Sorsdahl, Rodney Dawson

**Affiliations:** 1 Centre for Lung Infection and Immunity, Division of Pulmonology, Department of Medicine and UCT Lung Institute and South African MRC/UCT Centre for the Study of Antimicrobial Resistance, University of Cape Town, Cape Town, South Africa; 2 Centre for TB Research Innovation, University of Cape Town Lung Institute, George Road, Mowbray, Cape Town, 7925 South Africa; 3 The Desmond Tutu HIV Centre, Institute of Infectious Disease and Molecular Medicine, Cape Town, South Africa; 4 Alcohol, Tobacco and Other Drug Research Unit, South African Medical Research Council, Tygerberg, South Africa; 5 Addiction Psychiatry Division, Department of Psychiatry and Mental Health, University of Cape Town, Cape Town, South Africa; 6 Hannover Medical School, Carl-Neuberg-Str. 1, Hannover, Germany; 7 SA MRC Unit on Risk and Resilience in Mental Disorders, Department of Psychiatry and Neuroscience Institute, University of Cape Town, Cape Town, South Africa; 8 Alan J Flisher Centre for Public Mental Health, Department of Psychiatry and Mental Health, University of Cape Town, Cape Town, South Africa; 9 Division of Pulmonology, Department of Medicine, University of Cape Town Lung Institute, Cape Town, South Africa

**Keywords:** Tuberculosis, Adherence, Counselling, Substance use

## Abstract

**Background:**

Only 55% of multidrug-resistant tuberculosis (MDR-TB) cases worldwide complete treatment, with problem substance use a risk for default and treatment failure. Nevertheless, there is little research on psychotherapeutic interventions for reducing substance use amongst MDR-TB patients, in general, and on their delivery by non-specialist health workers in particular.

**Objectives:**

To explore the feasibility and acceptability of a non-specialist health worker-delivered 4-session brief motivational interviewing and relapse prevention (MI-RP) intervention for problem substance use and to obtain preliminary data on the effects of this intervention on substance use severity, depressive symptoms, psychological distress and functional impairment at 3 months after hospital discharge.

**Methods:**

Between December 2015 and October 2016, consenting MDR-TB patients admitted to Brewelskloof Hospital who screened at moderate to severe risk for substance-related problems on the Alcohol, Smoking and Substance Involvement Screening Test (ASSIST) were enrolled, and a baseline questionnaire administered. In the 4 weeks prior to planned discharge, trained counsellors delivered the MI-RP intervention. The baseline questionnaire was re-administered 3 months post-discharge and qualitative interviews were conducted with a randomly selected sample of participants (*n* = 10).

**Results:**

Sixty patients were screened: 40 (66%) met inclusion criteria of which 39 (98%) were enrolled. Of the enrolled patients, 26 (67%) completed the counselling sessions and the final assessment. Qualitative interviews revealed participants’ perceptions of the value of the intervention. From baseline to follow-up, patients reported reductions in substance use severity, symptoms of depression, distress and functional impairment.

**Conclusion:**

In this feasibility study, participant retention in the study was moderate. We found preliminary evidence supporting the benefits of the intervention for reducing substance use and symptoms of psychological distress, supported by qualitative reports of patient experiences. Randomised studies are needed to demonstrate efficacy of this intervention before considering potential for wider implementation.

**Trial registration:**

South African National Clinical Trials Register (DOH-27-0315-5007) on 01/04/2015 (http://www.sanctr.gov.za)

**Supplementary Information:**

The online version contains supplementary material available at 10.1186/s40814-020-00764-1.

## Introduction

It is estimated that 457,000 people developed multidrug-resistant tuberculosis (MDR-TB) in 2017 [1]. However, only 55% of MDR-TB cases were successfully treated to completion in 2015, with 21% of patients either lost to follow-up or with no outcome data. Treatment-related factors resulting in poor adherence to MDR-TB therapy and high default rates include excessive pill burden, injectable drug delivery, prolonged treatment duration and treatment-related side effects. In addition, behaviours such as drug use, cigarette smoking and hazardous or harmful patterns of alcohol use are also strongly associated with delays in diagnosis [2], more advanced disease [3], and poor treatment outcomes in MDR-TB [4–9].

The importance of substance use as a predictor of poor outcome in tuberculosis in general and MDR-TB in particular is recognised by the World Health Organisation. It has emphasised the need for a multisectoral approach to improving TB control, with one of its Sustainable Development Goals (SDGs) being to strengthen the prevention and treatment of substance use disorders including narcotic drug use, tobacco smoking and the harmful use of alcohol [1].

Surprisingly, given the considerable global effort to develop new drugs and regimens MDR-TB, there has been limited research on interventions to modify behaviours and improve treatment adherence in MDR patients with problematic substance use. Evidence-based strategies for addressing problem substance use amongst patients with TB may include methadone treatment for hospitalised opioid dependence [10], smoking cessation interventions through programmatic care [11], and screening and brief interventions to address hazardous and harmful alcohol use [12]. Few intervention studies have addressed substance use within the context of TB in South Africa: those that have been conducted are limited to alcohol and tobacco amongst patients with drug-susceptible TB who are being treated in outpatient settings [13]. Given the limited literature, further work is needed, particularly in low- and middle-income countries, where there are limited specialised mental health clinicians.

There is growing evidence that screening and brief interventions (SBI) for substance-related problems can be delivered effectively by non-specialised health workers [14]. These interventions are typically based on cognitive-behavioural and motivational interviewing techniques. A number of studies have been conducted in South Africa investigating the effectiveness of SBI amongst patients presenting to emergency centres [15], women receiving antenatal care [16], people receiving treatment for human immunodeficiency virus (HIV) and other chronic diseases in primary care settings [17–19], and hospitalised inpatients at a district-level hospital [20] and TB patients [12, 13].

Such interventions have not yet been evaluated in MDR-TB cohorts where the incidence of substance use is high. Unpublished data from our University of Cape Town Lung Institute drug-sensitive TB trial cohort showed that 17% of patients who actively deny drug use during informed consent have positive urine drug screens for substances such as cannabinoids, cocaine and amphetamines. A previous study from our setting showed that alcohol and other drug use were independently associated with MDR-TB treatment default [4]. The median time to default in this study was 257 days (or relatively early in the treatment phase of 2 years) and 25% of patients defaulted during initial hospitalisation either by refusing treatment or by absconding.

Psychosocial support in general, and addressing substance use and depression in particular, form an important but neglected area of patient-centred care in drug-resistant TB [21]. The psychosocial issues challenging MDR-TB patients are multifactorial and include difficulties caused by the illness itself, concomitant alcohol and substance use, the neuropsychiatric side effects of antituberculous drugs (like isoniazid, cycloserine or terizidone), as well as the stigma attached to the disease.

To address this gap, and as first steps towards intervening with this high risk population, we conducted an uncontrolled, single-arm feasibility test of a motivational interviewing and relapse prevention therapy (MI-RP) intervention for problem substance use. This paper reports on the (i) the feasibility of recruiting and retaining MDR-TB patients for a substance use intervention; (ii) preliminary information on the intervention’s substance use, mental health and TB outcomes; and (iii) participants’ perceptions of the acceptability of the intervention.

## Methods

### Trial design

An uncontrolled, single-arm feasibility test of a motivational interviewing and relapse prevention therapy (MI-RP) intervention for problem substance use.

### Setting

The study was conducted at Brewelskloof Hospital, a specialised TB hospital located in the town of Worcester in the Overberg District of the Western Cape Province, South Africa, which provides inpatient care for patients with drug-resistant TB. The hospital has 48 MDR-TB beds and admits ~ 150 patients per year with MDR-TB [4].

### Patients

Consecutive patients with MDR-TB (diagnosed on Xpert MTB/RIF assay (Cepheid, Sunnyvale, CA, USA) and/or liquid culture (BACTEC MGIT 960 system) [22]) admitted for the intensive phase of their treatment were recruited. Patients were eligible if they were ≥ 18 years of age and if they were at moderate to high risk for substance abuse, as measured by the Alcohol, Smoking and Substance Involvement Screening Test (ASSIST) [23]. The ASSIST consists of eight questions covering tobacco, alcohol, cannabis, cocaine, amphetamine-type stimulants, inhalants, sedatives, hallucinogens, opioids and ‘other drugs’. A risk score is provided for each substance, and scores are grouped into ‘low risk’, ‘moderate risk’ or ‘high risk’. The ASSIST takes approximately 5 to 10 min to administer. The ASSIST has been previously been validated for use in South Africa [24]. Patients with known psychiatric illness and/or significant medical comorbidities affecting insight, mobility or access to continued care were excluded from the study.

### Procedures

**Fig. 1:**
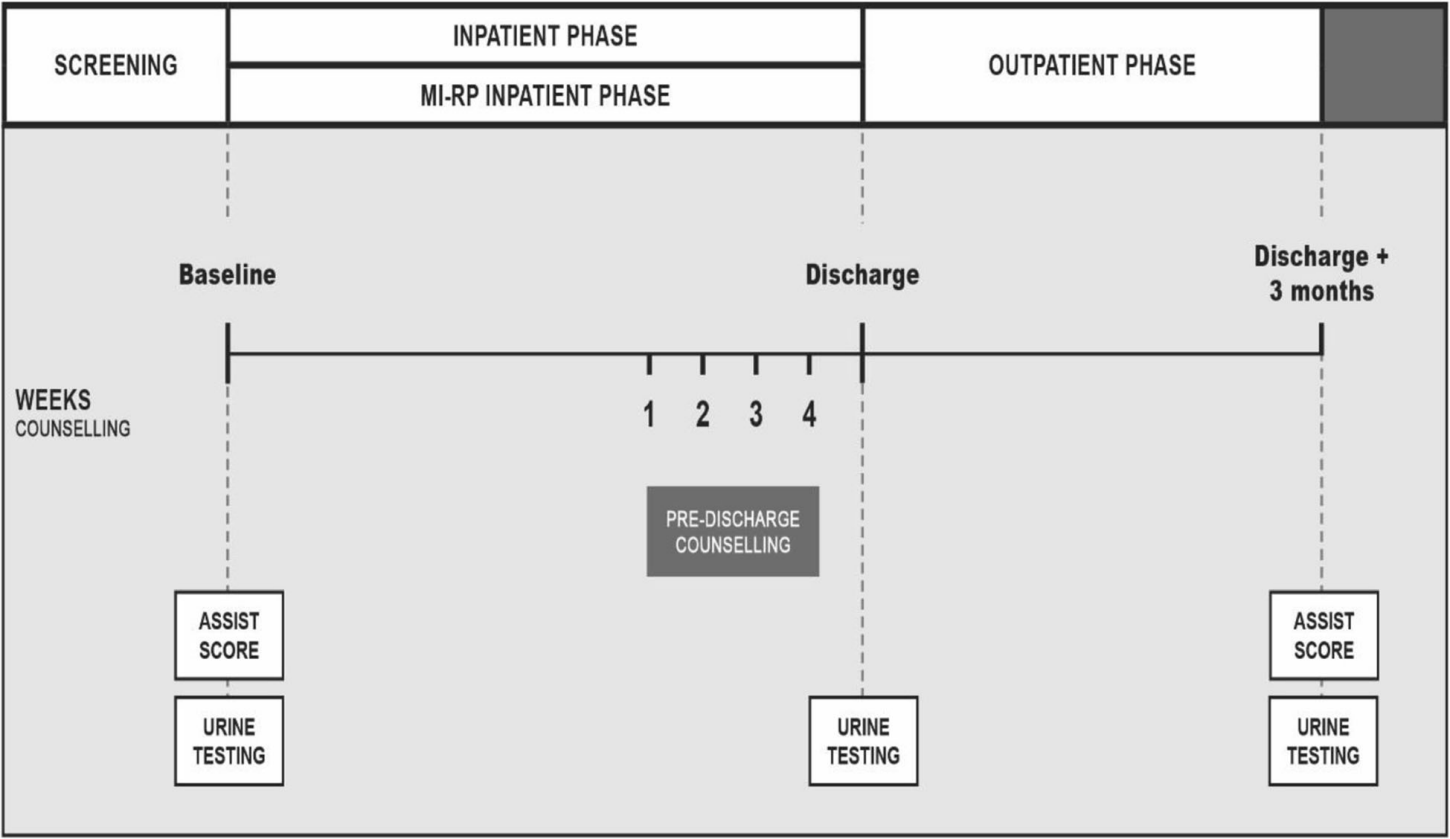
Diagram of study outline

After hospital admission, lay counsellors approached patients for study eligibility screening. After obtaining the patient’s consent, the counsellor administered the ASSIST. Low-risk substance users were thanked for their time and encouraged to maintain low risk usage, whilst patients scoring in the moderate and high risk range were invited to participate in the intervention. Patients who provided written consent were enrolled in the study and were asked to provide locator information. Field staff then administered a baseline questionnaire. Four weeks prior to their anticipated discharge date, patients received a motivational interviewing and relapse prevention (MI-RP) intervention in their preferred language (English, Afrikaans or Xhosa). Participants were contacted for a 3-month post-discharge follow-up assessment (3MFU) in which the baseline assessment was re-administered by a fieldworker not involved in the counselling (Fig. 1). MDR-TB treatment retention was recorded, and treatment outcomes (culture conversion, completion and cure) were obtained from the clinic register. Urine sample collection for illicit substances was performed at baseline, hospital discharge and at the 3MFU. The qualitative assessment involved in-depth interviews (guided by a semi-structured questionnaire) by ZdW in a random sample of 10 participants at the 3MFU end-point. Interviews were audiotaped and transcribed verbatim before the textual data was analysed qualitatively. Study procedures were overseen by the Human Research Ethics Committee of the University of Cape Town (UCT-HREC 004/2015). The clinical trial was registered with the South African National Clinical Trials Register (SANCTR) (DOH-27-0315-5007).

### Intervention

The MI-RP intervention consisted of 4 weekly sessions, each lasting between 45 and 60 min. Both MI and RP are considered evidence-based interventions for substance use disorders, and there is emerging evidence that the combination of these two approaches is effective for both substance use disorders [25] and helpful for medication adherence [26]. More specifically, motivational elements were included to build readiness for changing substance use and the RP content was included to help participants identify high-risk situations for substance use and external and internal triggers that may lead to thoughts and urges to use substances, and to teach them cognitive-behavioural strategies for managing these triggers and situations. During the intervention sessions, the counsellor provided feedback on the participants’ risk for substance-related harms, helped the participant set goals and identify barriers to change, and guided the participant in identifying high risk situations and triggers for substance use while teaching the participant techniques for managing both external and internal factors that place them at risk for relapse. Participants were provided with opportunities within the counselling sessions to practice these techniques. These techniques were also summarised in a written information booklet which included additional take-home activities to cement these new skills.

### Counsellor training and fidelity

Two counsellors were each trained to deliver the intervention in the patient’s preferred language; one spoke English or Afrikaans, whilst the other spoke isiXhosa. Both counsellors had at least a Bachelor qualification in either psychology or social work, and originated from the communities in the area. They received 40 h of training from BM and KS, followed by a proficiency test. Training included (i) substance use and the risks associated with substance use; (ii) using and scoring the ASSIST; (iii) ethics of research and importance of maintaining confidentiality and reporting adverse events; (iv) the intervention protocol; and (v) the process of managing distressed participants and referring patients for specialised care. To ensure intervention fidelity, counsellors completed a checklist to ensure all aspects of the intervention were provided. Additionally, all sessions were tape-recorded and random samples were selected for fidelity checking.

### Measures

To assess intervention feasibility, we recorded eligibility, recruitment, and treatment retention rates as well as study retention rates at follow-up. Substance use severity at baseline and 3MFU was measured using the ASSIST [23]. The following secondary outcomes were also measured at both time-points: Centre for Epidemiological Studies Depression Scale (CES-D), a measure of the most common symptoms of depression [27]; Fagerström Test for Nicotine Dependence (FTNF), a measure of the intensity of physical addition to nicotine [28]; EQ-5D 3L, a standardised instrument combining both descriptive and visual components as a measure of quality of life [29]; Sheehan Disability Scale (SDS), a measure of functional impairment in three inter-related domains: work, social and family life [30]; Self-Reporting Questionnaire 20 (SRQ20), a measure of psychological distress [31]. In addition, the Stages of Change Readiness and Treatment Eagerness Scale (SOCRATES), an experimental instrument designed to assess readiness for change [32], and the Multidimensional Scale of Perceived Social Support, (MSPSS) a measure of perceived social support using three subscales, namely: family, friends and significant others [33] were used. MDR-TB treatment outcome (culture conversion) was also recorded at the 3-month follow-up time point (which occurred at least 6 months after MDR-TB was initiated), and patients were followed up in the clinic register to determine final treatment outcome [34] (Supplementary file).

### Data analysis

Descriptive statistics were used to assess the number of potentially eligible patients (based on screeners), feasibility of recruitment (number and reasons for refusal to participate), participation (number of sessions attended) and completion rates. Frequency distributions and descriptive statistics were calculated for categorical and continuous variables. Paired sample *t* tests (for parametric data) and Wilcoxon signed rank tests (for non-parametric data) were used to assess initial effect of the intervention on the primary and secondary outcome variables. For patients reporting use of more than one substance, the substance for which they obtained the highest ASSIST score was compared. Statistical analyses were performed using GraphPad Prism (V·5·0, GraphPad Software, USA) and Stata (V.12.1, Stata Corp., College Station, TX, USA) [35]. Qualitative data analysis was conducted using the framework approach (familiarisation, identifying a thematic framework, indexing, charting, mapping and interpretation of data) [36], aided by NVivo 11.0 (a software programme). Initially, interview responses were read for emergent themes, which were then coded. To establish inter-coder reliability, each transcript was coded by two individuals who met to compare notes, establish a degree of agreement and resolve coding differences.

### Role of the funding source

The funders of the study had no role in study design, data collection, data analysis, data interpretation, or writing of the report. The corresponding author had full access to all the data in the study and had final responsibility for the decision to submit for publication.

## Results

### Patient flow and feasibility

**Fig. 2:**
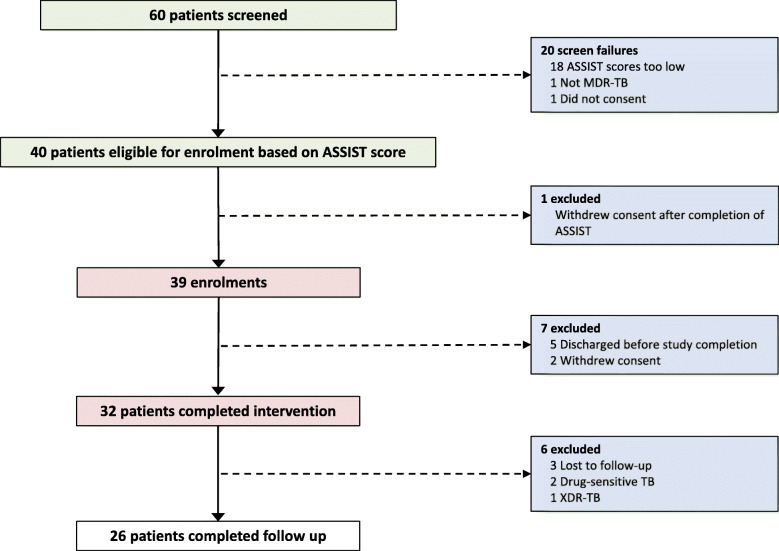
CONSORT diagram

During the study period, 60 consecutive inpatients were screened, of which 40 (70%) met substance use criteria for participation and were recruited to the intervention study (Fig. 2). 39/40 (97%) of the eligible patients received at least one counselling session; 32/40 (80%) completed all four counselling sessions and 26/40 (65%) completed the 3-month follow-up (overall study retention rate). In total, 14/40 recruited patients (33%) were excluded from the final analysis: 5 (13%) because they were discharged before the inpatient counselling intervention was completed, 3 (8%) because they were ultimately found not to have MDR-TB (one false-positive genotypic for rifampicin resistance, and two cases of extended drug resistance), 3 (8%) because they were lost to follow-up and 3 (8%) because they withdrew consent. One patient withdrew consent after the ASSIST score was administered but before any counselling sessions were administered, and two withdrew consent during the inpatient intervention.

### Sample description

The last column in Table 1 shows the demographic characteristics of the group of patients that completed the MI-RP intervention. There were no differences between the demographics of the overall sample and the groups of patients that were excluded or who completed the intervention (Table 1). The majority of patients were male (*n* = 14; 54%), with a mean (SD) age of 36.3 (10.6), and of mixed race or “coloured” (*n* = 20; 77%). [The term “coloured” refers to an ethnic group of people who possess some degree of sub-Saharan ancestry, but not enough to have been considered Black African during apartheid. It is a commonly used marker of race identity in South Africa]. Almost all patients had some school education (*n* = 25; 95%), with mean (SD) school grades completed of 7.8 (3); the majority were unemployed (*n* = 16; 62%) and lived in formal housing (*n* = 20, 77%). The mean (SD) ASSIST score was 19 (6.1), indicating moderate risk; only 2 (8%) patients were high risk (ASSIST score ≥ 27). Alcohol was the most commonly used substance, with 25 patients (96%) reporting alcohol use in the 3 months before enrolment; 5 patients (42%) reporting cannabis use, 1 (4%) reporting cocaine use, 3 (12%) reporting amphetamine use and 2 (8%) reporting inhalant use. Regular (either daily or several times per week) alcohol, cannabis and amphetamine use was reported in 16 (62%), 4 (16%) and 1 (4%) of patients, respectively. Four (15%) patients regularly used more than one substance.

**Table 1:** Differences between patients who completed the intervention and those who dropped out

Variables	Dropouts^a^ (*n* = 14)	Completed intervention (*n* = 26)
Age (years)		
Median (IQR)	28 (26–39)	36 (27–43)
Gender		
Males, *n* (%)	6 (43%)	14 (54%)
Education		
Any schooling, *n* %	13 (93%)	25 (97%)
Highest grade completed, median (IQR)	8 (6–9)	7 (6–11)
Housing		
Formal housing, *n* %	8 (57%)	20 (77%)
No. of people sharing house, med (IQR)	4 (3–6)	4 (3–7)
No. of rooms in house, med (IQR)	3 (1–5)	3 (2–4)
Employment		
Employed, *n* %	3 (21%)	10 (38%)
Unemployed,	11 (79%)	15 (58%)
Full-time student, *n* %	1 (3%)	1 (4%)
Smoking status		
Current smoking, *n* %	5 (36%)	13 (50%)
Previous smoking, *n* %	4 (29%)	7 (27%)
Never smoked, *n* %	5 (36%)	6 (23%)

^a^Fourteen patients excluded after enrolment: 5 (13%) because they were discharged before the inpatient counselling intervention was completed, 3 (8%) because they were ultimately found not to have MDR-TB (one false-positive genotypic for rifampicin resistance, and two cases of extended drug resistance), 3 (8%) because they were lost to follow-up and 3 (8%) because they withdrew consent

There were no statistically significant differences

### Preliminary effects

The ASSIST score decreased following the intervention (pre-intervention median (IQR) 17.5 (15–24) versus 6 (6–8)). Depressive symptomatology improved, with the median (IQR) CES-D score significantly lower post-intervention [23.5 (18–34) vs. 17 (13–22)]. Several other secondary measures also improved post-intervention (Table 2). There were also significant improvements in nicotine dependence, health status, functional impairment, psychological distress, readiness for change and perceived social support (Table 2). Only two patients (8%) had positive urine screening for a drug of abuse at follow-up. All patients that completed follow-up were adherent to treatment, culture-converted and were ultimately cured.

**Table 2:** Changes in primary and secondary outcomes in 26 patients completing the intervention

Questionnaire	Baseline Med (IQR)	Follow-up Med (IQR)
ASSIST^b^ (alcohol and substance use)		
Highest score^a^	17.5 (15–24)	6 (6–8)
Alcohol	17.5 (15–23)	6 (6–8)
Cannabis	0 (0–6)	0 (0–6)
Amphetamines	0 (0–0)	0 (0–0)
CES-D (depression)	23.5 (18–34)	17 (13–22)
FTND^c^ (nicotine dependence)	2 (2–4)	1 (1–2)
EQ-5D 3L (health status		
Descriptive elements	5.5 (5–7)	5 (5–5)
Visual analogue scale	70 (57–80)	90 (80–100)
SHS (disability)		
Work/school	0 (0–3)	0 (0–0)
Social life	2.5 (0–5)	0 (0–3)
Family life/home responsibilities	4 (0–7)	0 (0–3)
SRQ-20 (psychological distress)	8 (4–10)	3 (1–4)
SOCRATES (readiness for change)		
Recognition	20 (19–22)	15 (15–19)
Ambivalence	13 (11–15)	8 (4–10)
Taking steps	35 (32–39)	40 (38–40)
MDSPSS (social support)		
Significant other	8 (4–10)	3 (1–4)
Family	7 (6–7)	7 (6–7)
Friends	6 (4–6)	5 (4–6)
Total	6 (5–6)	6 (6–7)

*Abbreviations*: *ASSIST* Alcohol, Smoking and Substance Involvement Screening Test, a measure of problematic alcohol and drug use [23], *CESD-D* Centre for Epidemiological Studies Depression Scale, a measure of the most common symptoms of depression [27], *FTND* Fagerström Test for Nicotine Dependence, a measure of the intensity of physical addition to nicotine [28], *EQ-5D 3L*, a standardised instrument combining both descriptive (lower score indicates better health) and visual components as a measure of health outcome [29], *SDS* Sheehan Disability Scale, a measure of functional impairment in three inter-related domains: work, social and family life [30], *SRQ-20* Self-Reporting Questionnaire 20 (SRQ20), a World Health Organisation screening test for anxiety and depression disorders [31], *SOCRATES* Stages of Change Readiness and Treatment Eagerness Scale, an experimental instrument designed to assess readiness for change [32], and *MSPSS* Multidimensional Scale of Perceived Social Support, a brief self-report questionnaire with 12 items that subjectively measure perceived social support using three subscales, namely, family, friends and significant others (MSPSS) [33]

^a^Four (15%) patient regularly used more than one substance before the intervention; the substance with the highest ASSIST score was used

^b^No patient reported sedative, hallucinogen, opiate or methaqualone use

^c^Of the 11 smoking patients who scored > 1 on the Fagerström test at baseline

### Patients’ experience and acceptability

Three themes emerged from qualitative data that reflect patients’ perceptions of the acceptability of the counselling programme. The first theme highlights patients’ levels of motivation for addressing their substance use. The second theme describes the acceptability of the proposed counselling programme. The third theme describes recommendations for modifying the counselling programme.

### Motivation for behaviour change

High levels of motivation for addressing substance use were reported by all patients. Reasons put forward to why they agreed to receive counselling included “to want to resist it, to bury alcohol away forever” and “I wanted to quit alcohol, but I didn’t know how to do that”. This motivation for changing alcohol use seemed to go hand in hand with their health-related concerns and the desire to live a healthier life. This is illustrated by two patients who had the following to say about why they agreed to access counselling:

“I decided to participate in the study because I realised it was the right way to live your life, to let go of things that are wrong; like drugs, alcohol, and using syringes [Patient 58]”.

“I decided to take part because I didn't want to be like I was anymore. And the programme really helped me to stop doing the things I was doing before. [Patient 45]”.

### Acceptability of counselling programme

Patients reported positive experiences with the counselling programme, noting that “it was just so nice” and “I was happy all those times when I went for my sessions”. A few expressed that their stay in the hospital provided the space and time to reflect on their life and their health. The counselling sessions facilitated this reflection and ultimately their change in behaviour. As some patients reflected:

“I learned a lot from the sessions. I was always someone who liked to make trouble, do things like drinking and smoking, but now I choose to do things like taking a walk, gardening or playing football” [Patient 58].

“They helped me a lot. Number one, I no longer do alcohol. Even if a person is drinking, I don’t have that thing to say let me also take a sip [Patient 47]”.

There was consensus that the content, structure and delivery of the programme were acceptable. Patients could not identify any aspect that was redundant or not applicable to their needs. The counsellor delivering the programme content was acknowledged for her kindness, understanding, and ability to listen and explain the programme content clearly. These characteristics were conveyed by the following patients:

“I was a bit nervous at the beginning, but she made me feel very comfortable, she explained things to me so well. [Patient 34]”.

“I felt good because I could talk to her and she always understood what I was talking about. [Patient 33]”.

The written information booklet that accompanied the counselling sessions was considered highly valuable to patients, not only while they were receiving the counselling sessions in the hospital but following discharge as well. A few described using the booklet as a resource to refer to when they are facing challenges in their life. Several patients described how they were still referring to the booklet:

“I liked the part about experience, and what you are going to do when you leave the hospital, and writing every day about my experiences, what I did that day. [Patient 58]The things in the booklet still have value for me, I recently took out the booklet again, and just yesterday I was thinking about the booklet again, and I went through it and looked at the old work assignments I did. [Patient 37]”.

### Recommendations for modifying the counselling programme.

Although many patients thought that the duration of the intervention was appropriate, approximately half expressed that additional sessions would have been valuable. Unfortunately, whether these sessions should be conducted in hospital or after they were discharged into the community was not explored in the present study. The preference for additional sessions is expressed by the following patient:

“I think there should be more sessions. I enjoyed the counselling very much, I liked it and looked forward to it [Patient 37]”.

One of the challenges that some of the patients expressed during the counselling sessions was completing the homework tasks due to low levels of literacy. Four patients reported that they identified someone in the hospital, either another patient or a healthcare worker to assist them to complete the tasks.

## Discussion

This study is the first to examine an MI-RP intervention for problem substance use for hospitalised patients with MDR-TB had two main findings. First, our study demonstrates feasibility and acceptability of this intervention. Moderate retention rates indicate that it is possible to recruit, retain and follow-up patients with MDR-TB. Almost all eligible patients were willing to participate in a counselling intervention to reduce their substance use, demonstrating the acceptability of the intervention. This was reinforced by qualitative report, which emphasised that patients found the content, structure and delivery of the intervention acceptable. Over three-quarters of patients who consented to participate in MI-RP completed all four sessions, and the majority of patients expressed a desire for additional intervention sessions, which further supports the acceptability and perceived utility of this intervention. Second, findings from this small pilot study suggested that a MI-RP intervention shows preliminary outcomes that are promising. Following the intervention, there was a significant reduction in composite risk scores for substance use, with patients on average moving from moderate risk use to low risk use. There was also a significant reduction in depressive symptomatology and improvements in measures of psychological distress, functional impairment, nicotine dependence, readiness for change and perceived social support.

Acceptability and feasibility of this intervention is consistent with a range of other work on task-sharing in low- and middle-income countries [37, 38]. The use of counsellors was highly acceptable to patients, supporting the use of non-speciality health workers to deliver such an intervention. The feasibility of employing non-speciality workers rather than mental health professionals for this intervention has important positive implications for scalability in resource-limited environments such as South Africa.

Preliminary evidence of efficacy of a counselling intervention in improving substance use severity is also consistent with previous work, albeit in patients without TB [16, 20]. There are no studies of counselling interventions in MDR-TB that have focused specifically on substance use, and the few studies that have evaluated counselling interventions in drug-resistant TB have focused only adherence and treatment outcomes in outpatients [39–41]. Hospitalised patients are a particularly attractive target population for a substance abuse intervention because there is restricted availability and opportunity for substance use, and a planned intervention has the potential to prepare patients for discharge by preventing relapse and lifestyle management.

The finding that alcohol was the most commonly used substance is significant as it is one of the risk factors for MDR-TB [42]. Drug resistance may result from interruptions in drug-sensitive treatment resulting from alcohol use [43], and treatment default is also attributed to alcohol use [44]. There is an urgent need for effective intervention strategies to address alcohol abuse amongst the MDR-TB patients to bring about better treatment compliance.

Several limitations of this study deserve emphasis. First, the sample size was small, and there was no comparison group; further adequately powered randomised controlled trials are needed to confirm these findings. Second, data on patients not enrolled in or not completing the study intervention were not collected; improvements in substance use outcomes could represent regression to the mean or a non-specific effect. Improvements in depression and other measures could be reflective of response to antituberculous treatment and general improvement in health and are also confounded by the differences in setting between baseline and follow-up (inpatient vs. outpatient status). Previous studies have shown that psychological symptoms in MDR-TB are more severe at diagnosis and during the early stages of the disease [21]. Third, only illicit substances were able to be tested on urine; without a measure to confirm reductions in alcohol use, the treatment effect could not be objectively confirmed. Fourth, there was also a better than expected MDR-TB treatment outcome in the study group, with no treatment failures, perhaps indicating a sub-set of more impressionable, willing and adherent patients. Lastly, our preliminary findings may not be generalisable to all patients with MDR-TB; there has been a global move away from hospitalising patients with MDR-TB, and South Africa has decentralised and deinstitutionalised treatment since 2011 [45]; the study population therefore represents a relatively small proportion of the patients under care in the National Treatment Programme with MDR-TB.

## Conclusions

To the best of our knowledge, this study is the first to demonstrate that a counselling intervention delivered by non-specialist health workers to address substance use in MDR-TB is feasible and acceptable, and potentially promising for reducing substance use and improving mental health. However, as this was an uncontrolled, single-arm study, findings remain preliminary, and the efficacy of MI-RP for reducing problematic substance use will need to be studied. The evidence gained from this feasibility test will form the basis for future studies of task-sharing MI-RP-based interventions such as group MI-RP, outpatient MI-RP and nurse-driven MI-RP, which could be accessible to all MDR-TB patients.

## Supplementary Information

**Additional file 1.**

## Data Availability

The datasets used and/or analysed during the current study are available from the corresponding author on reasonable request.

## Abbreviations

3MFU: Three-month follow-up assessment
ASSIST: Alcohol, Smoking and Substance Involvement Screening Test
CES-D: Centre for Epidemiological Studies Depression Scale
FTNF: Fagerström Test for Nicotine Dependence
HIV: Human immunodeficiency virus
IQR: Interquartile range
MDR-TB: Multidrug-resistant tuberculosis
MI-RP: Motivational interviewing and relapse prevention
MSPSS: Multidimensional Scale of Perceived Social Support
SANCTR: South African National Clinical Trials Register
SBI: Screening and brief intervention
SD: Standard deviation
SDG: Sustainable Development Goals
SDS: Sheehan Disability Scale
SOCRATES: Stages of Change Readiness and Treatment Eagerness Scale
SRQ20: Self-Reporting Questionnaire 20
TB: Tuberculosis
UCT HREC: University of Cape Town Human Ethics Research Committee
