# Supplementary material for: Brief psychotherapy administered by non-specialised health workers to address risky substance use in patients with multidrug-resistant tuberculosis: a feasibility and acceptability study
Source: Pilot Feasibility Stud. 2021 Jan 19;7:28. doi: 10.1186/s40814-020-00764-1 (PMC7814702; doi:10.1186/s40814-020-00764-1)
Supplement: Supplementary file 1 — Additional file 1. [file 40814_2020_764_MOESM1_ESM.docx]

**Additional file**

This appendix has been provided by the authors to give readers additional information about their work.

Supplement to:

***A psychosocial intervention to address risky substance use and medication adherence amongst inpatients with multi-drug resistant tuberculosis in South Africa: a pilot study***

Gregory L. Calligaro, Zani de Wit, Jacqui Cirota, Catherine Orrell, Bronwyn Myers, Dan J. Stein, Katherine Sorsdahl and Rodney Dawson.

Corresponding author:

Professor Rodney Dawson

Head, Centre for TB Research and Innovation

University of Cape Town Lung Institute

George Road, Mowbray, 7925

Cape Town, South Africa

Tel: +27 21 650 1910 Fax: +27 86 240 2589

Email: [rodney.dawson@uct.ac.za](mailto:rodney.dawson@uct.ac.za)

Screening and assessment instruments:

1. The **Alcohol, Smoking and Substance Involvement Screening Test (ASSIST)** [1] is validated in several developing countries and is administered to assess the extent of problematic alcohol and drug use. A substance-use involvement score is calculated for each substance used in the preceding 3 months. In the case of multiple substances, only the highest score will be included in the total score. Urine sample testing for illicit substances will be used to validate these self-report measures.
2. The **Fagerström Test for Nicotine Dependence** [2] is a standard instrument for assessing the intensity of physical addiction to nicotine by evaluating the quantity of cigarette consumption, the compulsion to use, and grade of dependency. The total score obtained categorizes patients into low, low-moderate, moderate and high nicotine dependence groups.
3. The **Centre for Epidemiological Studies Depression Scale (CES-D)** [3] is designed to measure common symptoms of depression in the general population and consists of 20 self-rated items. Each item is rated on a four-point Likert scale, ranging from 0 (indicating no symptom presence) to 3 (indicating the presence of symptoms most of the time). Composite scale scores range from 0 to 60, with a score of 16 or higher signifying clinically meaningful depression.
4. The **Morisky Medication Adherence Scale (MMAS-8)** [4] is a good screening and monitoring tool for high-risk non-adherent patients used to assess adherence of the participants to their MDR-TB medication. Scores on the MMAS-8 range from 0 to 8, with scores of 3 or more reflecting low adherence to medication.
5. The **EQ-5D 3L** [5] is a standardized instrument combining both descriptive and visual components as a measure of health outcome. The descriptive component looks at 5 dimensions: mobility, self-care, usual activities, pain/discomfort and anxiety/depression with each dimension having 3 levels: no problems, some problems, extreme problems. The visual component records the respondent’s self-rated health on a visual analogue scale with endpoints labelled ‘the best health you can imagine’ and ‘the worst health you can imagine’. The selected digits for 5 dimensions are combined in a 5-digit number describing the respondent’s health state.
6. **Sheehan Disability Scale (SDS**) [6] assesses functional impairment in three inter-related domains: work, social and family life by using a 10-point visual analogue scale.
7. **Self-Reporting Questionnaire 20 (SRQ20)** [7] is a World Health Organisation screening test for anxiety and depression disorders. It is comprised of 20 questions.
8. **Stages of Change Readiness and Treatment Eagerness Scale (SOCRATES**) [8] is an experimental instrument designed to assess readiness for change. We have combined “substance use” in this questionnaire to include both alcohol and illicit substance use. Each item is rated on a five-point scale ranging from strongly disagree to strongly agree. The instrument yields three factorially-derived scale scores: recognition, ambivalence and taking steps and will be used to assess their motivation to change their behavior.
9. **Multidimensional Scale of Perceived Social Support (MSPSS)** [9] is a brief self-report questionnaire with 12 items that subjectively measure perceived social support using three subscales, namely: family, friends and significant others each rated on scale ranging from “very strongly disagree” to “very strongly agree.” Each item is scored between 1-7. The composite score obtained categorizes patients into low, moderate and high acuity of perceived social support.

**References**

1. WHO Assist Working Group: **The Alcohol, Smoking and Substance Involvement Screening Test (ASSIST): development, reliability and feasibility**. *Addiction* 2002, **97**(9):1183-1194.

2. Fagerstrom KO, Schneider NG: **Measuring nicotine dependence: a review of the Fagerstrom Tolerance Questionnaire**. *J Behav Med* 1989, **12**(2):159-182.

3. Radloff LS: **The CES-D Scale: A Self-Report Depression Scale for Research in the General Population**. *Applied Psychological Measurement* 1977, **1**(3):385-401.

4. Morisky DE, Ang A, Krousel-Wood M, Ward HJ: **Predictive validity of a medication adherence measure in an outpatient setting**. *J Clin Hypertens (Greenwich)* 2008, **10**(5):348-354.

5. Rabin R, Charro Fd: **EQ-SD: a measure of health status from the EuroQol Group**. *Annals of Medicine* 2001, **33**(5):337-343.

6. Sheehan DV, Harnett-Sheehan K, Raj BA: **The measurement of disability**. *Int Clin Psychopharmacol* 1996, **11 Suppl 3**:89-95.

7. Beusenberg M, Orley J: **A User's guide to the self reporting questionnaire (‎SRQ).** In: *World Health Organisation.* 1994.

8. Miller WR, Tonigan JS: **Assessing drinkers' motivation for change: The Stages of Change Readiness and Treatment Eagerness Scale (SOCRATES)**. *Psychology of Addictive Behaviors* 1996, **10**(2):81-89.

9. Zimet GD, Dahlem NW, Zimet SG, Farley GK: **The Multidimensional Scale of Perceived Social Support**. *Journal of Personality Assessment* 1988, **52**(1):30-41.
